# Effect of trimmed asparagus by-products supplementation in broiler diets on performance, nutrients digestibility, gut ecology, and functional meat production

**DOI:** 10.14202/vetworld.2022.147-161

**Published:** 2022-01-26

**Authors:** Manatsanun Nopparatmaitree, Marisa Nava, Verachai Chumsangchotisakun, Pornpan Saenphoom, Soranot Chotnipat, Warangkana Kitpipit

**Affiliations:** 1 Faculty of Animal Science and Agricultural Technology, Silpakorn University, Phetchaburi IT Campus, Cha-Am, Phetchaburi, 76120, Thailand; 2 Akkhraratchakumari Veterinary College, Walailak University, Nakhon Si Thammarat, 80160, Thailand; 3 One Health Research Center, Walailak University, Nakhon Si Thammarat, 80160, Thailand; 4 Food Technology and Innovation Research Center of Excellent, Walailak University, Nakhon Si Thammarat, 80160, Thailand.

**Keywords:** asparagus by-products, broiler, functional feed, functional meat, prebiotic

## Abstract

**Background and Aim:**

Trimmed asparagus by-products (TABP) is the resultant waste from asparagus possessing. TABP has fructans, such as inulins and fructooligosaccharide, which can be utilized as an alternative prebiotic. This study was conducted to examine the effect of TABP dietary supplementation on the productive performance, nutrient digestibility, gut microbiota, volatile fatty acid (VFA) content, small-intestine histology, and meat quality of broilers.

**Materials and Methods:**

A total of 320 1-day-old broiler chicks (Ross 308^®^) were raised under ambient temperature and assigned through a completely randomized design to one of four dietary treatments, with four replicates per treatment. The dietary treatments comprised corn-soybean basal diet supplemented with 0 (control), 10, 30, or 50 g/kg TABP. All birds were provided drinking water and feed *ad libitum* to meet the standard nutritional requirements of National Research Council for broiler chickens.

**Results:**

TABP supplementation to the broilers significantly increased the apparent ether extract, crude fiber, and gross energy digestibility (p<0.05). TABP supplementation significantly increased lactic bacteria and *Enterococcus* spp. numbers as well as acetic, propionic, butyric, and total VFA levels (p<0.01); on the other hand, it also significantly decreased *Salmonella* spp. and *Escherichia coli* contents in the cecum compared with the control group (p<0.01). Moreover, TABP supplementation increased villus height in the duodenum and jejunum (p<0.01), cryptal depth in the jejunum and ileum (p<0.01), and villus surface areas in the duodenum, jejunum, and ileum (p<0.01). Overall, 0-35 day TABP supplementation significantly increased the feed intake (p<0.01) and average daily gain of broilers (p<0.05), but not significantly affected the viability, productive index, and economic benefit return (p>0.05). The carcass characteristics, pH, color, and water holding capacity of the chicken meat between groups were not significantly different (p>0.05). All levels of TABP supplementation appeared to be a feasible means of producing broilers with the lower serum low-density lipoprotein cholesterol and triglyceride levels as well as atherogenic indices of serum compared with the control (p<0.05). Cholesterol contents and palmitic acid, oleic acid, saturated fatty acids, and Monounsaturated fatty acids levels decreased with an increase of TABP supplementation (p<0.05). Furthermore, TABP supplementation decreased atherogenic index (AI) and thrombogenicity index (TI) of meat (p<0.05).

**Conclusion:**

Supplementation of 30 g/kg TABP in broiler diet could enhance broiler performance and provide chicken meat with beneficial properties, with decreased AI and TI resulted from altered cholesterol and fatty acid profiles.

## Introduction

Diets could influence various functions of the body and improve the health status of livestock. Many nutrient groups are classified as functional feeds, including probiotics, prebiotics, synbiotics, and phytobiotics [1]. After 2006, the European Union announced the need to eliminate antibiotics in broiler feed to reduce the presence of antibiotic residues in meat products, which could affect drug resistance and consumer health [2]. This initiative resulted in the use of a popular functional feed in the broiler production industry and reductions in antibiotic resistance. Moreover, natural products with functional properties have been used in animal husbandry for growth and other indirect benefits. In addition, The World Health Organization predicted that cardiovascular disease will become the leading cause of death in 2030 and affect approximately 23.6 million people worldwide [3]. Functional food plays an important role in human life and can potentially promote optimal health status and reduce disease risk. Therefore, raising broiler on functional diets to produce functional broiler meat products is especially intriguing in the context of health-conscious dietary trends.

Agricultural countries generate a large amount of agricultural waste, which impacts the environment due to incineration, landfill, and allowing agricultural waste to rot and decompose naturally. As a result, proper waste disposal is a major challenge. Bio-circular-green economies strongly support research into high-potential, low-cost raw materials derived from agricultural waste for functional feeds. Asparagus is an attractive functional feed source because it is a popular Thai economic crop for domestic consumption and foreign export. Trimmed asparagus by-products (TABPs), which refer to asparagus parts that cannot be sold or processed during the quality selection phase of vegetable production, make up approximately 30-40% of the total harvest [4]. Asparagus root contains fructans and comprises approximately 25% of the fresh weight of the plant [5]. The amount of fructans in the edible portion of asparagus varies depending on the variety and could range from 0.5% to 2% (dry weight); asparagus also has high fiber contents, a distinct flavor, and several phytochemicals (e.g., vitamins, fructans, flavonoids, cinnamic acids, and saponins). TABP is an interesting material for use as a functional broiler feed due to its high fructan (e.g., inulin and fructooligosaccharide [FOS]) contents [6]. Fructans are very common prebiotics with structures consisting of short-chain and non-digestible carbohydrates [7]. Inulin and FOS are essential for beneficial microorganisms to survive in the digestive tract. The beneficial microorganism in the gut system can thrive with the assistance of inulin and FOS because they can be used as substrates for the survival and multiplication of probiotics in the lower gut region, where they act as symbiotic bacteria [8]. The previous research on broiler chickens showed that inulin and FOS supplementation can stimulate the gut fermentation of useful bacteria, such as bifidobacteria and lactobacilli, and limit the colonization of infective bacteria, such as *Salmonella* spp. and *Escherichia coli* [9]. Prebiotics can be fermented in the intestine by health-promoting bacteria to yield lactic acid and short-chain fatty acids (SCFAs) [10]. SCFAs are important energy sources in the intestine. Butyrate is a major SCFA metabolized by epithelial cells to provide energy for mucosal epithelial growth, improve the intestinal mucosal structure, and positively modify the gut microbiota [11]. Inulin and FOS can enhance the innate and acquired immune response [12], nutrient digestibility efficiency, and growth performance [13]. Furthermore, they can decrease serum cholesterol and improve the quality of chicken meat by stimulating probiotic fermentation to produce SCFAs [14] and bile salt hydrolase (BSH) during the hypocholesterolemic process [15]. The BSH can lower total cholesterol in the body [16]. The previous study showed that prebiotics and probiotics can decrease low-density lipoprotein (LDL) cholesterol and triglyceride levels [16], while increase high-density lipoprotein (HDL) cholesterol, and modify lipid compositions [17].

The researches on asparagus waste as prebiotic supplementation in livestock diets are limited. This study aimed to investigate the effect of TABP supplementation of broiler diets on the apparent nutrient digestibility, microbial ecology, small-intestine histology, production performance, carcass characteristics, and meat quality and fatty acid composition. The results could contribute to the current understanding of approaches to optimize digestive functions, nutrient utilization, and meat production by supplementing broiler diets with asparagus waste.

## Materials and Methods

### Ethical approval

The Animal Care Protocol Management and Review Committee of the Faculty of Animal Science and Agricultural Technology, Silpakorn University, approved the experimental protocol for this study (record no. ASAT SU0101/2562).

### Study period and location

This study was conducted from October to December 2019 (for animal husbandry) and from January to March 2020 (for laboratory) at Agricultural Technology Training and Transfer Center, Faculty of Animal Science and Agricultural Technology, Silpakorn University, Phetchaburi IT campus.

### TABP sample

TABP was collected from Hup-krapong (12.7775, 99.9096), Cha Am district, Phetchaburi Province, as part of The Royal Project of His Majesty the King of Thailand. TABP (90% root stock, 10% spear) were sliced and spread on a plastic sheet for 3 days before being oven-dried at 60°C for 3 days. Dried TABP sample was ground to a uniform size of 2 mm by pulverizing machine (RT-34, Chyun Tseh Industrial Co., Ltd., Republic of China). Furthermore, chemical composition of TABP was analyzed according to Association of Official Analytical Chemists (AOAC) [18], as well as FOS content was determined using thin-layer chromatography according to Reiffová and Nemcová’s [19]. Nutrient composition analyzed that TABP contains 86.80% dry mater, 18.50% crude protein, 0.61% ether extract, 37.62% crude fiber, 9.23% crude ash, 2175.23 kacl/kg, 0.10% calcium, 0.66% phosphorus, and 1.84% FOS.

### Experimental design, diets, and birds

A total of 320 (160 male and 160 female) 1-day-old Ross 308 broiler chicks with an average body weight of 40.46±1.25 g were obtained from a commercial hatchery (Farmmach Hatchery, Cha Am, Phetchaburi, Thailand). The birds were assigned through a completely randomized design to one of four treatments with four replicates (n=20 birds). The treatments applied were T1: Ration without TABP, T2: Ration+10 g/kg TABP powder; T3: Ration+30 g/kg TABP powder, and T4: Ration+50 g/kg TABP powder. The birds were housed in floor pens with new rice hulls in an open-sided house system. Newly hatched birds were brooded at 35°C and 60% RH for 10 days and then exposed to ambient temperature until the end of the experiments at 35 days. The broilers were vaccinated against Marek’s disease at the hatchery, Newcastle disease and infectious bronchitis at the farm on day 7, and Gumboro disease on day 14. All birds were offered *ad libitum* access to feed and water. The nutrient content of the diets used in the study corresponded to the needs of broilers raised in tropical climates. Throughout the experiments, the birds were fed mash diets for starters (1-21 days; 23% CP, 3200 kcal/kg) and growers/finishers (21-35 days; 20% CP, 3200 kcal/kg) according to the National Research Council (NRC) [20] as shown in Table-1.

**Table-1:** Ingredient composition and nutritive value of the experimental diet.

Experimental diet*	Starter diet (0-21 days)				Finisher diet (22-35 days)			
	
	T1	T2	T3	T4	T1	T2	T3	T4
Ingredient composition (%)								
Corn	49.50	48.50	49.50	49.50	46.92	46.92	46.92	46.92
Soybean meal (44%CP)	36.50	36.32	35.96	35.60	30.90	30.72	30.36	30.00
TABP^1^	-	1.00	3.00	5.00	-	1.00	3.00	5.00
Defatted rice bran	8.00	7.18	5.54	3.90	12.50	11.68	10.04	8.4
Rice bran oil	1.70	1.70	1.70	1.70	5.56	5.56	5.56	5.56
Limestone (CaCO_3_)	1.35	1.35	1.35	1.35	1.20	1.20	1.20	1.20
DCP (18%P)	2.10	2.10	2.10	2.10	1.90	1.90	1.90	1.90
Choline Chloride-L	0.03	0.03	0.03	0.03	0.03	0.03	0.03	0.03
NaCl	0.14	0.14	0.14	0.14	0.23	0.23	0.23	0.23
DL-Methionine (99%)	0.34	0.34	0.34	0.34	0.28	0.28	0.28	0.28
L-lysine (98.5%)	-	-	-	-	0.22	0.22	0.22	0.22
L-Threonine (98.5%)	0.15	0.15	0.15	0.15	0.06	0.06	0.06	0.06
Premi×^2^	0.20	0.20	0.20	0.20	0.02	0.02	0.02	0.02
Total	100.00	100.00	100.00	100.00	100.00	100.00	100.00	100.00
Nutritive value from laboratory analysis (%)								
Dry matter	90.35	90.31	91.02	90.67	91.13	90.76	90.87	90.49
Crude protein	23.56	23.91	23.71	13.57	20.49	20.36	20.41	20.33
Ether extract	5.39	5.23	5.60	5.00	5.51	5.30	5.52	5.63
Crude fiber	4.26	4.19	4.51	4.49	3.54	3.79	4.87	4.90
Ash	8.11	7.93	7.36	6.35	6.82	7.10	6.61	7.31
Gross energy (Kcal/kg)	4079.30	4033.60	4012.30	4073.90	4089.60	4069.90	4148.90	4082.00

* T1: Ration without TABP, T2: Ration+10 g/kg TABP powder; T3: Ration+30 g/kg TABP powder, and T4: Ration+50 g/kg TABP powder. ^1^TABP=Trimmed asparagus by-products. ^2^Each one kilogram of vitamin-mineral premix contained 20.02 MIU of retinal palmitate, 9.10 MIU of cholecalciferol, 136.50 g of DL-3-tocopheryl acetate, 5.46 g of phylloquinone, 5.46 g of thiamine, 14.56 g of riboflavin, 27.30 g of Ca-D-pantothenate, 7.28 g of pyridoxine, 109.20 g of niacin, 3.64 g of folic acid, 29.12 mg of cobalamin, 237.00 mg of D-biotin, 120 g of manganese, 3.00 g of selenium, 1000 mg of zinc, 160.00 mg of copper, 400.00 mg of ferrous, 12.50 g of iodine. TABP=Trimmed asparagus by-products

### Apparent nutrient digestibility

The apparent digestibility of nutrients was calculated using the indicator method of Fenton and Fenton [21]. Thirty-two 1-day-old chicks were randomly placed in metabolic cages (two birds/cage) for feces collection to study digestibility. All birds in each cage had free access to water and feed containing 0.3% chromium oxide (Cr_2_O_3_) from days 0 to 18 to adopt to the diets and metabolic cages. Feces collection was then performed over 3 days (days 19-21). Feathers and other contamination were carefully removed, and the excreta collected in each cage with 3% H_2_SO_4_ over 2 days was pooled and stored at –20°C. Excreta samples were thawed and dried at 60°C for 48 h, ground through a 0.5 mm sieve, and then stored in airtight plastic containers for analysis according to Mountzouris *et al*. [22] and Ghayour-Najafabadi *et al*. [23]. Feed and feces samples were analyzed in the laboratory for dry matter, organic matter, crude protein, crude fiber, ether extract, gross energy, and Cr_2_O_3_ contents according to the method of AOAC [18]. Apparent dry matter and nutrient digestibility were calculated according to Zewdie [24].

### Cecal microbiota, volatile fatty acid (VFA) contents, and small-intestine histomorphology

On day 21, four birds (i.e., two males and two females) from each pen with the closest weight to the mean body weight were sacrificed by cervical dislocation. Cecum contents were sampled to count the number of microorganisms and then packed in ice buckets for laboratory analysis. Exactly 1.5 g of cecum content samples was decomposed in sterile water (1:1 g/vol) in a screw-capped tube and sampled at –20°C for VFA analysis according to Khattak *et al*. [25]. Duodenal, jejunal, and ileal samples measuring approximately 2.5 cm in length were cut and rinsed with saline solution to remove the digesta, according to Shang *et al*. [12]. The samples were fixed in 10% neutral-buffered formalin, according to Gava *et al*. [26]. The numbers of lactic acid bacteria, *Enterococcus*, *E. coli*, and *Salmonella* in 1 g of cecal fluid samples were counted. The fluid samples were then diluted ten-fold using a culture technique with selective media De Man, Rogosa and Sharpe agar (Difco, USA) + 0.02% NaH_3_ + 0.05% L-cystine hydrochloride monohydrate, Eosin methylene blue agar (HiMedia, India), and m-Enterococcus agar (HiMedia) according to McDonald *et al*. [27], Horn *et al*. [28], and Schillinger and Holzapfel [29]. The number of microorganisms was transformed using the base 10 log algorithm [30].

VFA concentrations were determined from the cecum contents collected from each experimental group. Sample preparation for VFA content determination was performed according to Walugembe *et al*. [31]. The sample solution was injected into a gas chromatograph (HP 5890 Series II GC; Agilent J&W; 30 m×0.535 mm×1.00 micron HP-FFAP column) to detect acetic, propionic, and butyric acids; 4-methylvaleric acid (Alfa Aesar, United Kingdom) was used as the internal standard, and a flame-ionization detector was used for measurement. The VFA composition of the samples was then compared with the standard solution according to Khattak *et al*. [25].

For histological studies, samples were dehydrated, cleared, and embedded in paraffin in the laboratory using standard histological procedures. Six-micrometer-thick sections were cut, placed on glass slides, stained with hematoxylin and eosin, and examined under a light microscope, according to Wang *et al*. [2]. The histomorphology of the small intestine was assessed using an Olympus BX 50 optical microscope at 20× magnification and analyzed using the Motic Images 2.0 multi-language program [32]. Samples of 10 villi per slide were evaluated to determine villus height, width, and cryptal depth, according to Wang *et al*. [2]. The surface area of a villus was calculated {(¶)×Villus width/2)×Villus height)} according to Sakamoto *et al*. [33].

### Productive performance and economic returns

Chickens were reared for 35 days and observed daily. The body weight, feed intake, and mortality of the birds were recorded on days 21 (i.e., the end of the starter phase) and 35 (i.e., the end of the grower/finisher phase). Average daily feed intake, average body weight gain (BWG), average daily gain (ADG), feed conversion ratio (FCR; feed/gain), and viability were calculated as indicators of production performance following the formulas described by Marcu *et al*. [34]. Production index (PI) was calculated following the formula of Barbosa *et al*. [35]. Economic return indicators, such as feed cost per gain (FCG), salable bird return (SBR), net profit per bird (NPR), and return on investment (ROI) were calculated according to the formulas of Ojediran *et al*. [36].

· PI=(ADG (kg)×livability/FCR)×100
· FCG=(FCR×feed cost×BWG)
· SBR =(Price of live chicken×Body weigth)
· NPR =(SBR–FCG)
· ROI =(NPR/FCG)×100.

### Hematology, carcass, meat quality, and meat fatty acid composition

The blood samples used in this study were collected from 35-day-old broilers; two males and two females per pen were used. Blood samples were collected from the wing vein into tubes with EDTAK3 for plasma sampling according to Seifi *et al*. [37]. Blood was also sampled into non-EDTAK3 tubes. The serum in these samples was harvested and stored at –20°C for biochemical analysis according to Dev *et al*. [38]. Hematocrit, red blood cell (RBC), white blood cell (WBC), heterophils (H), and lymphocytes (L) were determined, and the H/L ratio was calculated according to Reisinger *et al*. [39]. Serum cholesterol and triglyceride levels were determined using an automatic hematology testing machine (Advia 120, Bayer, Tarrytown, NY, USA) through an enzymatic colorimetric method (i.e., the CHOD-PAP method) according to Zhao *et al*. [13]. Serum atherogenic indices, including cardiac risk ratio (CRR) and atherogenic coefficient, and plasma atherogenic index (AI) were calculated according to Dev *et al*. [38].

· CRR=Total cholesterol/HDL cholesterol
· AC=(Total cholesterol−HDL cholesterol)/HDL cholesterol.

Body weight at slaughter was calculated on day 35 as the average weight of 6 h fasted birds in each experimental group at the time of slaughter. Four birds (i.e., two males and two females per pen were used) were slaughtered by mechanical stunning and then bled near the occipital bone and atlas. Carcasses were individually wrapped in plastic bags and chilled for 24 h in a 5°C chilling room. The carcasses were subsequently weighed and cut into commercial parts. Carcass percentage, chilled carcass percentage, and cutting percentage were calculated following the method of Faria *et al*. [40]. pH values at 45 min and 24 h were determined by inserting electrodes into the breast meat samples and using a contact pH meter system (Model 205, Testo AG, Lenzkirch, Germany).

Breast meat (without skin) color was measured from the surface of the samples with a chromameter (Minolta 410, Japan), which was standardized with a white tile. Color was expressed in terms of CIE values for lightness (L*), redness (a*), and yellowness (b*); these values were calculated from three readings at different positions according to Petracci *et al*. [41]. Hue angle and chroma were calculated according to Pathare *et al*. [42]. Water holding capacity (WHC), such as drip loss, cooking loss, trawling loss, and roasting loss, was determined according to Barbosa *et al*. [35].

Breast meat samples were collected to determine total cholesterol levels through the C45,994.10 method according to AOAC [18]. Individual fatty acid contents were determined by isolating and analyzing each fatty acid through gas chromatography (HP6890; Agilent, Waldbronn, Germany). Fatty acids were quantified from their retention times in standard solutions by mass spectrometry (HP5973, Agilent) according to the method of Lepage and Roy [43]. Lipid quality index was calculated from fatty acid data, such as iodine value and ratio of saturated fatty acids to unsaturated fatty acids (SFA/USFA), according to Zhai *et al*. [44]. AI, D-9 desaturase (16), and D-9 desaturase (18) indices were evaluated according to He *et al*. [45]. Thrombogenicity index (TI) and ratio of hypocholesterolemic to hypercholesterolemic fatty acid (h/H ratio) were determined according to Loponte *et al*. [46].

### Statistical analysis

The experimental data were evaluated by analysis of variance (ANOVA) with a completely randomized block design using the statistical model Y_ij_=μ+T_i_+e_ij_, where μ is the general mean, T_i_ is the effect of treatment (i=control and TABP supplementation at levels of 10, 30, and 50 g/kg), and e_ij_ is the random error associated with observation Y_ij_. ANOVA revealed significant differences, Tukey’s honestly significant test was performed as described by Steel and Torrie [47] using R version 3.5.1 software as described by the R Core Team [48].

## Results

This study assessed the productive performance of broiler chickens over 22-35 days and 0-35 days of rearing periods revealed that TABP supplementation at levels of 30 and 50 g/kg led to higher feed intake, BWG, and ADG compared with the control group (p<0.05). Increases in level of TABP supplementation had a linear effect on total feed intake and daily feed intake (p<0.01). BWG and ADG increased with increasing TABP supplementation levels in broiler diets in a linear manner (p<0.05), but TABP supplementation did not affect survival rates or the PI in all age groups of broiler chickens (p>0.05). When the economic return of feeding broilers with all levels of TABP supplementation was calculated, the FCG, SBR, NPR, and ROI of chicken production were not statistically different across all experimental groups (p>0.05), as presented in Table-2.

**Table-2:** Effects of TABP supplementation in broiler diets on productive performance and economic benefit return.

Parameters	Level of TABP supplementation in broiler diets (g/kg)				SEM	p-value	Trend Analysis

	0	10	30	50			
Feed intake (kg/bird/day)							
0-21 days	1.29	1.22	1.31	1.39	0.18	0.06	NS
22-35 days	2.52^C^	2.62^BC^	2.85^AB^	3.12^A^	0.04	<0.01	L
0-35 days	3.81^C^	3.84^BC^	4.16^AB^	4.51^A^	0.05	<0.01	L
Average daily feed intake (g/bird/day)							
0-21 days	61.68	58.29	64.42	66.31	0.85	0.06	NS
22-35 days	179.72^C^	186.91^BC^	203.48^AB^	222.75^A^	3.01	<0.01	L
0-35 days	108.89^C^	109.74^BC^	118.85^B^	128.88^A^	1.36	<0.01	L
Body weight gain (kg/bird/day)							
0-21 days	0.87	0.81	0.83	0.84	0.01	0.40	NS
22-35 days	1.09^b^	1.38^a^	1.29^a^	1.39^a^	0.03	0.02	L
0-35 days	1.95^b^	2.19^a^	2.12^a^	2.23^a^	0.04	0.02	L
Average daily gain (g/bird/day)							
0-21 days	41.30	38.64	39.60	39.99	0.52	0.40	NS
22-35 days	77.61^b^	98.64^a^	92.11^a^	99.40^a^	2.09	0.02	L
0-35 days	55.82^b^	62.64^a^	60.60^a^	63.75^a^	0.73	0.02	L
Feed conversion ratio (Feed/Gain/day)							
0-21 days	1.49^A^	1.51^AB^	1.57^C^	1.66^D^	0.01	<0.01	L
22-35 days	2.33	1.92	2.21	2.24	0.06	0.19	NS
0-35 days	1.95	1.76	1.96	2.02	1.36	0.09	NS
Viability (%) (day)							
0-21 days	98.33	100.00	100.00	100.00	0.42	0.44	NS
22-35 days	91.23	8.25	96.49	100.00	1.52	0.27	NS
0-35 days	89.47	98.25	96.49	100.00	1.24	0.07	NS
Productive index (day)							
0-21 days	271.47	255.44	251.74	240.97	14.37	0.13	NS
22-35 days	204.43	346.82	270.23	296.31	17.08	0.09	NS
0-35 days	256.60	353.76	299.04	315.71	11.14	0.08	NS
Economic benefit return (USD)							
Feed cost per gain	2.135	1.973	2.201	2.270	1.04	0.06	NS
Salable bird return	2.258	2.312	2.312	2.312	0.43	0.44	NS
Net profits return per bird	0.123	0.340	0.111	0.042	1.16	0.09	NS
Return of investment (%)	5.88	17.78	5.17	2.00	2.00	0.09	NS

^a,b^Mean with symbol with in same row differ significantly different (p<0.05),

A,B Mean with symbol with in same row differ significantly different (p<0.01), SEM=Standard error of mean, NS=Not significantly different (p>0.05), L=Linear. TABP=Trimmed asparagus by-products

The digestibility of nutrients and discovered that broilers provided a diet supplemented with TABP at levels of 10, 30, and 50 g/kg have higher ether extract, crude fiber, and gross energy digestibility than the control group (p<0.05). TABP supplementation at 10 g/kg also led to higher organic matter digestibility than the control and TABP 50 g/kg group (p<0.01). Increases in TABP supplementation in broiler diets had a quadratic effect on ether extract, crude fiber, and gross energy digestibility (p<0.05), as shown in Table-3.

**Table-3:** Effects of TABP supplementation in broiler diets on apparent nutrient digestibility.

Parameters (%)	Level of TABP supplementation in broiler diets (g/kg)				SEM	p-value	Trend analysis

	0	10	30	50			
Dry matter	83.77	88.75	85.97	85.12	0.84	0.21	NS
Crude protein	80.44	81.29	81.08	85.09	0.99	0.30	NS
Ether extract	92.28^c^	96.62^ab^	97.97^a^	95.55^ab^	0.81	0.04	Q2
Organic matter	86.78^B^	91.26^A^	87.82^AB^	85.52^B^	0.41	<0.01	Q2
Gross energy	87.44^d^	91.29^ab^	92.08^a^	90.09^bc^	0.60	0.02	Q2
Crude fiber	77.89^c^	82.60^b^	87.05^a^	80.79^b^	0.29	0.01	Q2

^a,b^Mean with symbol with in same row differ significantly different (p<0.05),

A,B Mean with symbol with in same row differ significantly different (p<0.01), SEM=Standard error of mean, NS=Not significantly different (p>0.05), and Q2=Quadratic. TABP=Trimmed asparagus by-products

Broilers provided a diet with TABP supplementation at levels of 10, 30, and 50 g/kg revealed greater numbers of cecal lactic acid bacteria (e.g., *Lactobacillus* and *Bifidobacterium*) and *Enterococci* than the control group (p<0.01). TABP supplementation at levels of 10, 30, and 50 g/kg also showed the lower numbers of *Samonella* spp. in the cecum compared with the control group (p<0.01). The number of *E. coli* in the cecum of broilers given functional feed supplemented with TABP was lower than that of the control group, with significant differences in TABP groups at 30 and mg/kg. Increasing the level of TABP supplementation in broiler diets appeared to increase the number of lactic acid bacteria (e.g., *Lactobacillus* and *Bifidobacterium*) and *Enterococcus* spp. in the cecum (quadratic, p<0.01). The numbers of *Samonella* spp. and *E. coli* in broiler chickens fed functional feed containing TABP supplementation decreased as the level of TABP supplementation increased in a quadratic (p<0.01) and linear (p<0.01) manner, respectively, as shown in Table-4. In addition, broilers fed diet supplemented with TABP at levels of 10, 30, and 50 g/kg had higher levels of acetic, propionic, butyric, and total VFA in the cecum than the control group (p<0.01). Total volatile fatty, acetic, propionic, and butyric acids in the cecum increased with the level of TABP supplementation in a quadratic manner (p<0.01), as shown in Table-4.

**Table-4:** Effects of TABP supplementation in broiler diets on cecal microbiota and volatile fatty acids content.

Parameters	Level of TABP supplementation in broiler diets (g/kg)				SEM	p-value	Trend analysis

	0	10	30	50			
Cecal microbiology (Log10 colony-forming units/mL)							
Lactic acid bacteria*	11.35^B^	11.94^A^	12.36^A^	12.06^A^	0.26	<0.01	Q2
*Enterococci*	6.70^B^	7.22^A^	7.18^A^	7.27^A^	0.16	<0.01	Q2
*E. coli*	8.17^A^	7.73A^B^	7.33^B^	7.37^B^	0.23	<0.01	L
*Salmonella*	3.73^A^	3.29^B^	3.20^B^	3.15^B^	0.07	<0.01	Q2
Volatile fatty acids (µmol/mL)							
Total volatile fatty acid	67.69^B^	84.53^A^	80.15^A^	82.59^A^	1.36	<0.01	C
Acetic acid	48.30^B^	57.20^A^	54.48^AB^	56.44^A^	1.18	<0.01	C
Propionic acid	9.05^B^	11.05^A^	10.40^A^	10.48^A^	0.17	<0.01	C
Butyric acid	9.50^B^	11.50^A^	10.74^A^	11.00^A^	0.18	<0.01	C

* *Lactobacillus* and *Bifidobacteria*, ^a,b^Mean with symbol with in same row differ significantly different (p<0.05), SEM=Standard error of mean, NS=Not significantly different (p>0.05), L=Linear, Q2=Quadratic, C=Cubic. TABP=Trimmed asparagus by-products

The small-intestinal histomorphology of broilers fed diet with TABP supplementation revealed that TABP supplementation at a level of 30 g/kg of diets resulted in greater duodenal villus height (quadratic, p<0.01), surface areas (linear, p<0.01), and height per cryptal depth ratio (quadratic, p<0.01) compared with the control group. Furthermore, broilers given functional feed with TABP supplementation at levels of 10, 30, and 50 g/kg had higher jejunal villus heights, widths, and surface areas compared with the control group. These parameters increased with TABP in a quadratic manner (p<0.01), as shown in Table-5.

**Table-5:** Effects of TABP supplementation in broiler diets on small intestinal histomorphology.

Parameters	Level of TABP supplementation in broiler diets (g/kg)				SEM	p-value	Trend analysis

	0	10	30	50			
Duodenum							
Villus height (mm)	1.42^C^	1.58^AB^	1.65^A^	1.51^BC^	0.05	<0.01	Q2
Villus width (mm)	0.14^AB^	0.13^B^	0.14^AB^	0.15^A^	0.01	<0.01	Q2
VSA (mm^2^)	0.61^B^	0.65^AB^	0.70^A^	0.69^A^	0.03	<0.01	L
Cryptal depth (mm)	0.22	0.24	0.22	0.23	0.01	0.104	NS
VH:CD	6.56^B^	6.67^AB^	7.48^A^	6.73^A^	0.03	<0.01	Q2
Jejunum							
Villus height (mm)	1.11^B^	1.26^A^	1.28^A^	1.24^A^	0.04	<0.01	Q2
Villus width (mm)	0.14^B^	0.16^A^	0.16^A^	0.15^A^	0.01	<0.01	Q2
VSA (mm^2^)	0.47^B^	0.64^A^	0.64^A^	0.61^A^	0.03	<0.01	Q2
Cryptal depth (mm)	0.21^B^	0.23^AB^	0.24^A^	0.22^AB^	0.01	<0.01	Q2
VH:CD	5.30	5.62	5.41	5.56	0.26	0.36	NS
Ileum							
Villus height (mm)	0.76	0.85	0.87	0.81	0.05	0.10	NS
Villus width (mm)	0.14	0.13	0.13	0.12	0.01	0.43	NS
VSA (mm^2^)	0.24^B^	0.33^A^	0.34^A^	0.33^A^	0.01	<0.01	Q2
Cryptal depth (mm)	0.15^C^	0.16^B^	0.17^A^	0.17^AB^	0.01	<0.01	Q2
VH:CD	5.293	5.296	5.029	5.243	0.27	0.51	NS

^A,B^Mean with symbol within same row differ significantly different (p<0.01), SEM=Standard error of mean, NS=Not significantly different (p>0.05), L=Linear, and Q2=Quadratic, VSA=Villus surface area, VH:CD=Villus height: Crypt depth. TABP=Trimmed asparagus by-products

Table-6 shows that the dressing percentage, cutting percentage, pH, color (L*, a*, and b*), and WHC of broilers provided a diet at all levels of TABP supplementation were not significantly different from those of the control group (p>0.05). In addition, broilers provided diet with TABP supplementation at levels of 10, 30, and 50 g/kg also had lower serum LDL cholesterol, triglycerides, and atherogenic indices of serum (cardiac risk ration and atherogenic coefficient) than broilers in the control group (p<0.05). TABP supplementation at 30 and 50 g/kg levels led to lower serum triglycerides compared with the controls (p<0.05). LDL cholesterol, triglycerides, and atherogenic indices of serum in broiler chickens decreased linearly as the level of TABP supplementation increased (p<0.05). TABP supplementation did not affect other hematological parameters, such as RBC concentration, RBC count, WBC count, H, L, and H/L ratio (p>0.05), as shown in Table-7.

**Table-6:** Effects of TABP supplementation in broiler diets on carcass and meat quality.

Parameters	Level of TABP supplementation in broiler diets (g/kg)				SEM	p-value	Trend Analysis

	0	10	30	50			
Carcass and cutting percentage (%)							
Thai carcass	84.02	85.65	83.89	83.53	0.71	0.73	NS
Dressing percentage	75.95	77.45	76.96	76.34	0.43	0.64	NS
Chilled percentage	74.43	75.90	75.42	74.82	0.42	0.63	NS
Breast	25.20	29.65	28.57	29.58	0.28	0.42	NS
Fillets	4.56	5.12	4.78	5.21	0.12	0.34	NS
Wing	10.88	10.90	10.90	11.47	0.17	0.57	NS
Thigh	16.81	18.08	18.06	16.79	0.32	0.34	NS
Drum strict	11.01	11.58	11.20	11.29	0.17	0.69	NS
Head	6.41	6.81	6.09	6.03	0.25	0.69	NS
Shank	3.35	3.32	3.56	3.42	0.11	0.86	NS
Skeletal	18.91	17.21	20.73	18.39	0.21	0.02	NS
Internal organ	11.82	10.22	11.89	12.32	0.23	0.12	NS
Meat quality							
pH 0	6.21	6.18	6.32	6.29	0.37	0.59	NS
pH 24	5.92	5.89	5.90	5.99	0.14	0.48	NS
Color at 24 h after chilled storage at 4°C							
Lightness (L*)	55.87	52.50	56.80	55.63	0.66	0.19	NS
Redness (a*)	0.36	0.54	0.55	0.47	0.17	0.45	NS
Yellowness (b*)	9.76	9.54	10.07	10.31	0.43	0.92	NS
Chroma	0.001	0.003	0.003	0.002	0.00	0.03	NS
Hue angle	1.53	1.51	1.52	1.53	0.10	0.17	NS
Water holding capacity (%)							
Drip loss	4.55	4.11	4.38	4.57	0.25	0.91	NS
Cooking loss	21.75	23.04	21.39	22.43	0.86	0.90	NS
Trawling loss	10.01	9.00	11.66	6.69	0.88	0.31	NS
Roasting loss	21.06	20.29	18.23	19.94	0.77	0.63	NS

SEM=Standard error of mean, NS=Not significantly different (p>0.05). TABP=Trimmed asparagus by-products

**Table-7:** Effects of TABP supplementation in broiler diets on blood parameter and serum biochemistry.

Parameters	Level of TABP supplementation in broiler diets (g/kg)				SEM	p-value	Trend analysis

	0	10	30	50			
Blood parameter							
White blood cell (10^9^/mm^3^)	15.93	11.76	18.40	30.66	3.07	0.23	NS
Lymphocyte (%)	54.67	60.67	49.33	50.33	4.14	0.76	NS
Heterophile (%)	35.33	34.67	42.33	44.00	3.86	0.77	NS
H/L ratio	0.66	0.61	1.01	1.09	0.17	0.69	NS
Red blood cell (×10^6^/mm^3^)	1.67	2.00	1.67	2.40	0.12	0.20	NS
Hemoglobin (g/dL)	13.00	13.33	13.67	13.73	0.21	0.58	NS
Hematocrit (%)	22.67	26.33	22.00	34.33	2.02	0.19	NS
Serum biochemistry (mg/dL)							
Cholesterol	178.67	182.33	197.00	173.67	2.12	0.57	NS
High-density lipoprotein cholesterol	119.00	124.33	121.00	125.00	3.03	0.88	NS
Low-density lipoprotein cholesterol	53.00^A^	45.33^B^	46.33^B^	37.33^C^	0.33	0.04	L
Triglyceride	60.33^A^	63.67^A^	57.33^B^	57.00^B^	0.48	0.02	L
Atherogenic indices of serum							
Cardiac risk ratio	1.56^A^	1.46^B^	1.48^B^	1.40^B^	0.02	0.02	L
Atherogenic coefficient	0.56^A^	0.46^B^	0.48^B^	0.40^B^	0.02	0.02	L

^A,B^Mean with symbol within same row differ significantly different (p<0.01), SEM=Standard error of mean, NS=Not significantly different (p>0.05), L=Linear

The results of the present experiment demonstrated that the level of TABP supplementation in broiler diets lowers cholesterol contents in chicken meat in a linear manner (p<0.01). TABP supplementation at levels of 30 and 50 g/kg resulted in the lower SFA contents in meat than the control group (p<0.01). The results also revealed that broilers provided diet with TABP supplementation at levels of 10, 30, and 50 g/kg had low levels of palmitic, oleic, and monounsaturated fatty acids (MUFAs) in their meat compared with the control. TABP supplementation decreased the iodine value and omega-9 fatty acid content of chicken meat in a linear manner (p<0.01) but had no effects on omega-3 and omega-6 contents in (p<0.05), as indicated in Table-8.

**Table-8:** Effects of TABP supplementation in broiler diets on fatty acid profile in meat.

Parameters	Level of TABP supplementation in broiler diets (g/kg)				SEM	p-value	Trend analysis

	0	10	30	50			
Cholesterol (g/100 g)	72.50^A^	72.37^A^	71.01^AB^	68.25^B^	1.49	0.01	L
Fatty acid profile in meat(g/100 g)							
Myristic acid	0.01	0.01	0.01	0.01	0.00	0.00	NS
Palmitic acid	0.48^A^	0.38^B^	0.36^B^	0.32^B^	0.04	<0.01	L
Palmitoleic acid	0.08	0.07	0.06	0.06	0.06	0.42	NS
Stearic acid	0.10	0.10	0.13	0.14	0.01	0.07	NS
Vaccenic acid	0.03	0.03	0.03	0.03	0.04	0.69	NS
Oleic acid	0.62^A^	0.52^B^	0.48^B^	0.41^C^	0.03	<0.01	L
Linoleic acid	0.24	0.02	0.22	0.22	0.02	0.71	NS
Linolenic acid	0.01	0.01	0.01	0.01	0.00	0.00	NS
Eicosenoic acid	0.01	0.01	0.01	0.01	0.00	0.00	NS
Arachidonic acid	0.01	0.01	0.02	0.01	0.03	0.67	NS
∑SFA	0.63^A^	0.53^AB^	0.51^B^	0.47^B^	0.05	<0.01	L
∑MUFA	0.75^A^	0.64^B^	0.59^B^	0.52^C^	0.03	<0.01	L
∑PUFA	0.26	0.26	0.25	0.24	0.02	0.84	NS
∑Omega 3	0.01	0.01	0.01	0.01	0.00	0.00	NS
∑Omega 6	0.25	0.25	0.24	0.23	0.02	0.84	NS
∑Omega 9	0.63^A^	0.53^B^	0.49^B^	0.42^C^	0.03	<0.01	L
Quality of fat in meat							
SFA/USFA ratio	0.63	0.59	0.60	0.62	0.06	0.76	NS
Iodine value	1.08^A^	0.98^B^	0.93^BC^	0.86^C^	0.04	<0.01	L
n3/n6 ratio	0.04	0.04	0.04	0.04	0.00	0.75	NS
Atherogenic index	0.55^a^	0.47^b^	0.48^b^	0.47^b^	0.04	0.04	Q2
Δ-9 desaturase (16) index	12.87^B^	15.94^A^	14.72^AB^	15.81^A^	0.14	0.04	Q2
Δ-9 desaturase (18) index	81.20^A^	79.26^A^	78.06^AB^	74.27^B^	1.97	<0.01	L
Thrombogenicity index	1.12^a^	1.03^b^	1.04^b^	1.05^b^	0.02	0.04	Q2
h/H ratio	1.83^b^	1.97^a^	1.97^a^	1.99^a^	0.03	0.03	Q2

^a,b^Mean with symbol within same row differ significantly different (p<0.05), ^A,B^Mean with symbol within same row differ significantly different (p<0.01), SEM=Standard error of mean, NS=Not significantly different (p>0.05), L=Linear, and Q2=Quadratic. TABP=Trimmed asparagus by-products

The effect of diet supplemented with TABP on the health indices of chicken meat was demonstrated in this study. Broilers given functional feed supplemented with TABP at levels of 10, 30, and 50 g/kg had a lower AI than the control group (quadratic, p<0.05). The Δ-9 desaturase (16) and Δ-9 desaturase (18) indices of meat from broiler chickens fed TABP at doses of 10, 30, and 50 g/kg were higher than those of control broilers (p<0.05). However, broilers provided diet supplemented with TABP at levels of 10 and 30 g/kg revealed a Δ-9 desaturase (18) index of meat similar to that of the control group (p>0.05). TABP supplementation increased the Δ-9 desaturase (16) index; for TI a decrease is shown in Table-7 and h/H ratio of chicken meat in a quadratic manner (p<0.05), as indicated in Table-8.

## Discussion

The results of this study revealed that feeding TABP prebiotics to broilers could increase their ether extract, organic matter, gross energy, and crude fiber digestibility. Huang *et al*. [7] reported that asparagus contains high levels of FOS and inulin. These substances are classified as prebiotics because they help beneficial bacteria survive and grow in the gut [49]. Beneficial bacteria, also known as gut local probiotics, aid in the digestion and utilization of nutrients in monogastric animals [50]. Carbohydrate digestibility increased in the cecum because, as the main fermentation site in chickens, it contains a large number of microorganisms [51]. The digestive enzymes of monogastric animals are unable to digest FOS. Probiotics in the hindgut, such as lactic acid bacteria (e.g., *Bifidobacterium* spp. and *Lactobacillus* spp.) and *Enterococcus* spp., can digest FOS completely and produce gas, lactic acid, and SCFAs through carbohydrate fermentation [52]. The findings of the present experiments are consistent with those of Yun *et al*. [53], who found that prebiotic-treated broilers have higher dry matter digestibility than controls, as well as those of Huang *et al*. [7], who suggested that prebiotic and probiotic supplementation can improve nutrient digestion and absorption, leading to improved chicken performance. Meng *et al*. [54] discovered that supplementing prebiotics derived from oligosaccharides can improve the dry matter and protein digestibility of broilers.

This study found that supplementation of broiler feed with TABP increases VFA production and the number of beneficial microorganisms, such as lactic acid bacteria (e.g., *Lactobacillus* and *Bifidobacterium*) and *Enterococcus*, in the guts of broiler chickens relative to those in the control group. TABP supplementation could also reduce the numbers of *Samonella* spp. and *E*. *coli* compared with the control. According to Józefiak *et al*. [51], the prebiotics beta-glucan and inulin could increase the yield of short-chain VFAs (e.g., acetic acid, propionic acid, and butyric acid) in the gut through microbial oligosaccharide fermentation. Short-chain VFAs are important for the physiological processes of the intestinal microflora and can help improving gut health by modulating the microbial ecology [55]. Shang *et al*. [10] reported the prevalence of unique bacteria in broilers fed FOS supplements, such as *Akkermansia* (a mucin-degrading bacterium), *Janthinobacterium* and *Butyrivibrio* (butyrate-producing bacteria), *Coprococcus* (a butyrate-producing bacterium), and *Paludibacter* (a propionate-producing bacterium). FOS supplementation can increase the numbers of these bacteria at the epithelial wall of the ileum, improve intestinal immunity, and increase the membrane absorption area of nutrients [10,56,57]. These phenomena may inhibit some pathogenic bacteria and reduce the colonization of organisms such as *Salmonella* and *Campylobacter*. The crop, gizzard, and duodenum contain similar microorganisms up to 99% lactobacilli [58], resulting in an acidic ecology that is unsuitable for harmful bacterial growth, development, and division. Increases in fermentation activity and VFA content could be correlated with increased acidity, which results in pathogenic inhibition effects and increased nutrient digestibility [59]. Buclaw [60] described the ability of *Bifidobacterium* and *Lactobacillus* spp. to produce natural antibiotic substances with a broad spectrum of action, such as lactocin, helveticin, covaxin, nisin, and indocin. Furthermore, bacteria can produce bacteriocin, which inhibits the growth of *E. coli*; *Bifidobacterium*, and *Lactobacillus* can produce organic acids, that is, lactic acid and acetic acid, to suppress pathogenic pathogens in the gastrointestinal tract, that is, *Salmonella*, *Campylobacter*, and *E. coli* [61].

According to Ahmed *et al*. [62], increases in VFA may be attributed to the presence of inulin and oligosaccharides affect the reduction of pathogenic microorganisms in the cecum [10]. FOS can help maintain a healthy digestive environment by increasing the number of *Bifidobacterium* or decreasing the number of *E. coli* in the digestive tract. The findings of the present study are consistent with the results of many previous studies demonstrating the potential applications of prebiotics. Csernus and Czeglédi [63] found that high levels of FOS supplementation could boost microorganisms’ production of VFA, leading to improvement of host’s gut ecology. Biochemical studies and microbiological cultures showed that dietary FOS supplementation could increase gut fermentation, increase VFA production, stimulate the growth of beneficial bacteria, such as bifidobacteria and lactobacilli, and inhibit the growth of pathogenic microorganisms, such as *Salmonella* spp., *Clostridium perfringens*, and *E. coli* in broilers [64]. Supplementation with 0.25% FOS and 0.05% MOS or the antibiotic avilamycin could reduce the numbers of *C. perfringens* and *E. coli*; treatment with 0.25% FOS and 0.25% MOS also demonstrated a direct effect on the increase and diversity of lactobacilli in the ileum [65]. Geier *et al*. [66] reported that broilers fed 5 g/kg FOS show increased numbers of microorganisms in the ileum than the control group. The increased abundance of *Lactobacillus* and *Bifidobacterium* in the intestinal microflora of broilers fed prebiotics observed in the present study is similar to the results of Gaggìa *et al*. [67]. Some potential mechanisms that could explain the health benefits of prebiotics in altering the gut microbiota include competitive exclusion of pathogens [68], production of antimicrobial factors [69], stimulation of the immune system [70], and development of intestinal morphology [71].

The findings of this work indicated that broilers fed prebiotics from TABP have higher duodenal villus heights, widths, and surface areas compared with control birds because the epithelial cells of the small intestine use SCFAs produced by microbial fermentation as an energy source to stimulate the development and to increase the integrity of the intestinal mucosa. Increases in the number of beneficial microorganisms could reduce the number of harmful microorganisms directly affecting the villi; some harmful microorganisms, for example, produce toxins, such as botulinum toxin from *Clostridium botulinum* that destroy villus cells. Ahmed *et al*. [62] described the primary mechanism of prebiotics in promoting the growth of lactic acid-producing bacteria and their effect on increasing the concentration of SCFAs (e.g., acetic, propionic, and butyric acids), which are an important source of energy for colon cells and stimulate the intestines [72]. SCFAs provide energy to cell membranes and indirectly lower the cecum pH to prevent pathogen growth and increase mineral uptake [71]. Butyric acid is an important source of energy for epithelial cells and helps to suppress the inflammatory response by inhibiting pro-inflammatory cytokines [73]. According to Eeckhaut *et al*. [73], butyric acid provides energy to epithelial cells and resists the inflammatory response induced by pro-inflammatory cytokines.

Acetic acid is used as a solidifier in the formation of fats and cholesterol, while propionic acid is used as a solidifier in gluconeogenesis; the latter also inhibits fatty acid and lipid synthesis. Over 200 non-starch polysaccharide-degrading enzymes and pathways involved in the production of SCFAs have been discovered in the metagenomic analysis of the cecal microbiota [74]. The present research was consistent with the previous research demonstrating the potential of prebiotics in broiler diets to develop intestinal mucosal structures and villus heights [75] and improves gastrointestinal health and strength [49]. Considering the potential ability of prebiotics from dietary TABP supplementation to increase digestibility, improve intestinal ecology, and enhance the intestinal morphology, such supplementation may help improve feed utilization efficiency and growth performance of broilers.

The findings of the present study clearly demonstrated the effect of TABP supplementation into broiler diets on feed intake, weight gain, and no significant differences for PI in Table-1. These findings are consistent with the results of Kim *et al*. [65], who found that 0.5% FOS prebiotic supplementation increases ADGs compared with the control group; however, the authors also found that FCR and survival rates are similar across all experimental groups. Józefiak *et al*. [51] reported that beta-glucan and inulin could increase BWG and FCR. The current study also confirmed that TABP prebiotic supplementation does not affect carcass characteristics and meat quality, which is consistent with the findings of Abdel-Hafeez *et al.*, [76] who reported that prebiotic supplementation in broiler diets does not affect carcass, breast meat, and visceral percentages, including the liver, heart, and small intestine. Guaragnia *et al*. [77] indicated that inulin supplementation with probiotics does not affect the L*, a*, and b* color values, shear force values, and WHC of meat.

Dietary USFAs help lowering cholesterol, which is associated with coronary heart disease. PUFAs are associated with lower plasma LDL levels and the total cholesterol ratio [78]. The present study highlights the effect of TABP prebiotic supplementation on serum LDL cholesterol and triglyceride levels, as well as total cholesterol contents in chicken breast meat. The findings are consistent with those of the previous studies describing the lowering of serum cholesterol by supplementation with dietary oligosaccharides and [79] or *Spirulina platensis* [80,81]. Aktimur *et al*. [82] reported that the use of probiotics, prebiotics, and symbiotic lower total cholesterol, LDL cholesterol, and triglycerides in rats with high blood cholesterol levels. *Lactobacillus plantarum* LS/07 reduced total and LDL cholesterol, while *L. plantarum* Biocenol LP96 reduced triglycerides and very low-density lipoprotein without affecting serum HDL cholesterol and hepatic lipids [83]. A substantial body of evidence supports the assumptions about the mechanisms of prebiotic and probiotic supplementation in diets in cholesterol reductions.

The previous research described a mechanism of bacterial cholesterol absorption involving the binding of cholesterol to the bacterial cell wall [3] and then to the phospholipid bilayer membrane of probiotic cells [84]. Shehata *et al*. [85] discovered that growing bacterial cells deposit large amounts of cholesterol and that sonication stimulates cholesterol production in *Bifidobacterium breve* ATCC 15700 cells by over 40%. However, the binding of cholesterol to growing probiotic cells is highly stable. *In vitro* experiment revealed that the amount of cholesterol removal decreased when probiotics were grown. This finding suggests that cholesterol is removed not only by live probiotics during maturation but also by dead cells [86]. A compelling explanation for the reduction of cholesterol associated with bile formation has also been presented. According to Wang *et al*. [87], lactic acid bacteria may lower blood cholesterol by stimulating bile acid excretion in the stool, which increases the deconjugation and fecal excretion of bile acids [88]. This mechanism is believed to be an indirect means for prebiotics to lower cholesterol. The reduction in cholesterol is most likely due to the combination of pro- and prebiotics involved in the production of BSH, which catalyzes the breakdown of conjugated bile salts into unconjugated bile salts, such as glycol- and tauro-bile acids. The latter are absorbed less extensively than conjugated bile salts, resulting in greater bile excretion through the feces [86].

Dietary fiber inhibits bile reabsorption into the liver by increasing bile excretion, which results in a decrease in bile in the liver and stimulation of bile synthesis via the enzyme 7-hydroxylase. In addition, soluble fatty acids can inhibit cholesterol synthesis and stimulate bile production, affecting cholesterol reduction in the blood [89]. Acetic acid is converted to acetyl-coA, which acts as a precursor for cholesterol biosynthesis in the liver, and butyric acid is involved in the oxidation of mitochondrial fatty acids into acetyl-coA products [90]. Butyric acid inhibits the synthesis of cholesterol in the liver and serves as an energy source for human colon epithelial cells. Propionic acid inhibits fatty acid synthesis in the liver, reduces the rate of triacylglycerol secretion, and lowers overall cholesterol synthesis levels, leading to lower blood cholesterol levels [33]. According to Mistry *et al*. [91], increased acetic and butyric acid contents could be observed in the manure of inulin-treated animals and *in vitro* addition of propionic acid to hepatocytes reductase activity. This finding is consistent with the results of Kim *et al*. [92], who reported that SCFAs inhibit 3-hydroxy-3-methyl-glutaryl-coenzyme A (HMG CoA) reductase activity. Ooi [16] described the mechanism of conversion of cholesterol to coprostenol by probiotic cholesterol reductase and the inhibition of HMG CoA reductase by probiotics. HMG CoA is associated with a cholesterol synthesis pathway [89]. When cholesterol is reduced in the liver, HMG CoA reductase stimulates the synthesis of cholesterol and LDL receptors in the liver and the transport of cholesterol from the bloodstream to liver cells, resulting in decreases in blood cholesterol levels and cholesterol accumulation.

The present experiments showed that TABP supplementation could reduce the contents of palmitic acid, oleic acid, SFAs, and MUFAs in broiler chickens but had no effect on the latter’s omega-3 and omega-6 fatty acids. These findings are consistent with those of previous studies. Kalavathy *et al*. [93] found that probiotic supplementation is effective in lowering monounsaturated, oleic, and SFAs. Bonos *et al*. [94] demonstrated that the fatty acid composition of meat can be altered by microorganisms in the gastrointestinal tract because these microorganisms can hydrogenate USFAs to SFAs. Probiotics could also reduce the oleic acid contents of rat liver [95] and chicken breast [96]. Kalavathy *et al*. [93] revealed that *Lactobacillus* supplementation of broiler feed does not affect the poly-USFA contents of the chicken meat when compared with controls. Hossain *et al*. [97] found that herbs and probiotics increase arachidonic acid, DHA, and PUFA levels and decrease omega-6 fatty acids in breast meat. Kim *et al*. [65] found that prebiotic supplementation can increase linoleic acid contents but decrease arachidonic acid contents in chicken breast meat; no effect on PUFA levels was observed. Stearic and SFA proportions decreased whereas linoleic and PUFA contents increased in breast and thigh meat of broilers fed probiotics and cassava extracts [98]. A definitive mechanism to explain the effect of dietary probiotics on the regulation of palmitic acid contents in chicken breast meat has been proposed. Rodrigues *et al*. [99] reported that oligosaccharides with structures similar to those of FOS and inulin affect fatty acid levels to different extents because of differences in expression level and/or oligosaccharide-degrading enzyme activity Gomaa [100] discovered a link between consumer’s blood cholesterol and dietary levels of C16:0 and C14:0; specifically, C14:0 fatty acids induced greater increases in blood cholesterol compared with C16:0 fatty acids, and the latter had no effect on LDL and HDL changes. The findings of this study implied that changes in fatty acid ratio in egg yolks could result in lower cardiovascular disease risk factors (e.g., TI) and Ross *et al*. [101] discovered that probiotics could decrease the AI of functional foods. The present experiment results demonstrated that prebiotic supplementation could increase the metabolic disease inhibitory index (i.e., Δ-9 desaturase index) value and h/H ratio of chicken meat. Salah *et al*. [102] similarly found that probiotics increase the h/H ratio in animals; according to the findings of this study, the chicken meat obtained from broilers supplemented with APT as a dietary prebiotic, is suitable for use in functional food.

## Conclusion

Supplementation of broiler functional feed with TABP may increase the apparent digestibility of ether extract, crude fiber, and gross energy. TABP supplementation increased the yield of short-chain VFA in the hind gut, which promoted the proliferation of lactic acid bacteria and *Enterococcus* spp., and decreased the numbers of *Salmonella* spp. and *E. coli*. Broilers fed with TABP diet showed increased villus height and crypt of Lieberkühn depth of the duodenum, jejunum, and ileum, which could contribute to increased FI and ADG without changes in the carcass and meat quality. Supplementation of broiler diet with TABP reduced total cholesterol and triglyceride in serum, atherogenic indices of serum, as well as total cholesterol, palmitic acid, oleic acid, SFA, and MUFAs in breast meat. The addition of TABP to the broiler diet could also reduce AI and TI and increase the D-9 desaturase (16) index and h/H ratio of broiler meat, suggesting the resultant meat products as a healthy food. The results demonstrated that supplementation of 30 g/kg TABP optimally improves broiler performance and meat production. This research contributes to the guideline of using TABP to resolve the problem of agricultural waste, improve by-product value addition and broiler meat product development as functional foods to promote consumer health.

## Authors’ Contributions

MN: Conceptualized, data collection and analysis, and drafted the manuscript. MNa and VC: Husbandry and data collection. PS: Laboratory and data collection. SC: Review manuscript and editing. WK: Data analysis. All authors read and approved the final manuscript.
